# Evaluating the impact of patients' online access to doctors' visit notes: designing and executing the OpenNotes project

**DOI:** 10.1186/1472-6947-12-32

**Published:** 2012-04-13

**Authors:** Suzanne G Leveille, Janice Walker, James D Ralston, Stephen E Ross, Joann G Elmore, Tom Delbanco

**Affiliations:** 1 Division of General Medicine and Primary Care, Beth Israel Deaconess Medical Center, Harvard Medical School, Boston, USA; 2 College of Nursing and Health Sciences, University of Massachusetts Boston, Boston, USA; 3 Group Health Cooperative, Center for Health Studies, Seattle, USA; 4 Division of General Internal Medicine, University of Colorado, Denver, USA; 5 Department of Medicine, University of Washington School of Medicine, Harborview Medical Center, Seattle, USA

**Keywords:** Patient access to records, Electronic health records, Primary care physicians, Internet, Medical records, Medical informatics, Patient participation

## Abstract

**Background:**

Providers and policymakers are pursuing strategies to increase patient engagement in health care. Increasingly, online sections of medical records are viewable by patients though seldom are clinicians' visit notes included. We designed a one-year multi-site trial of online patient accessible office visit notes, OpenNotes. We hypothesized that patients and primary care physicians (PCPs) would want it to continue and that OpenNotes would not lead to significant disruptions to doctors' practices.

**Methods/Design:**

Using a mixed methods approach, we designed a quasi-experimental study in 3 diverse healthcare systems in Boston, Pennsylvania, and Seattle. Two sites had existing patient internet portals; the third used an experimental portal. We targeted 3 key areas where we hypothesized the greatest impacts: beliefs and attitudes about OpenNotes, use of the patient internet portals, and patient-doctor communication. PCPs in the 3 sites were invited to participate in the intervention. Patients who were registered portal users of participating PCPs were given access to their PCPs' visit notes for one year. PCPs who declined participation in the intervention and their patients served as the comparison groups for the study. We applied the RE-AIM framework to our design in order to capture as comprehensive a picture as possible of the impact of OpenNotes. We developed pre- and post-intervention surveys for online administration addressing attitudes and experiences based on interviews and focus groups with patients and doctors. In addition, we tracked use of the internet portals before and during the intervention.

**Results:**

PCP participation varied from 19% to 87% across the 3 sites; a total of 114 PCPs enrolled in the intervention with their 22,000 patients who were registered portal users. Approximately 40% of intervention and non-intervention patients at the 3 sites responded to the online survey, yielding a total of approximately 38,000 patient surveys.

**Discussion:**

Many primary care physicians were willing to participate in this "real world" experiment testing the impact of OpenNotes on their patients and their practices. Results from this trial will inform providers, policy makers, and patients who contemplate such changes at a time of exploding interest in transparency, patient safety, and improving the quality of care.

## 

Providers and policymakers are pursuing many strategies to increase the engagement of patients in promoting health and managing illness. As the general trend toward transparency accelerates, providers with electronic medical records (EMRs) increasingly allow patients to view online laboratory results, medication lists, and other parts of the medical record. However, while patients nationwide have the right to view their medical records, providers rarely share clinicians' visit notes proactively with their patients.

Offering visit notes to patients stirs concerns among doctors and their staff. Will the many patients inundate their doctors' offices with telephone and e-mail queries, placing excessive demands on already overburdened doctors and their staffs? Will patients be distressed or confused by what they read? Or would such access lead to more involvement in care, more sense of control, greater knowledge, and improved adherence to the plan of care? How would patients view comments about their mental health, likelihood of developing a malignancy, habits with alcohol, or efforts to control their weight?

Whatever the views of doctors or patients, in the presence of rapidly changing societal norms such change toward greater transparency will almost certainly occur in coming years. On the cusp of this change, we sought to begin to answer some important questions and concerns through the OpenNotes study, using a simple, one step intervention. We made all visit notes between consenting primary care physicians (PCPs) and their patients readily available online through secure patient Internet portals. We hypothesized that after a one year trial of OpenNotes, patients and PCPs would want it to continue. And we hypothesized further that OpenNotes would not lead to significant disruptions for doctors and their staffs.

In designing this trial, which would involve both a demonstration and evaluation of OpenNotes, we weighed the strategy of a standard experimental design against an effort that would focus on a large scale intervention involving multiple and highly diverse sites, each of which required a somewhat different intervention. We chose the latter approach in order to mount a "real world" experiment involving a large number of patients and PCPs in a one-year demonstration of OpenNotes. The many PCPs who declined participation in the intervention and their patients would thereby serve as the comparison groups for the trial. Basing our trial in the primary care environment in 3 different settings and geographical locations, our primary purpose was to assess PCPs and patients attitudes and experiences with OpenNotes. Secondly, we sought to gather information from a variety of sources in order to determine in a comprehensive way how the use of OpenNotes affects patient behavior and doctors' practices.

Crafting a careful evaluation of this intervention, which many viewed as potentially disruptive, across very different health care systems required both extensive planning and creative strategies to address the many differences in the populations and technologies across the 3 sites. With the hope that our unusual design might help inform other studies focusing on the evaluation of innovations in the practice of medicine, we describe in this paper the methods we developed and implemented. Also, we present initial results from our recruitment and implementation of OpenNotes.

## Methods/Design

### Settings

Although we specifically targeted primary care in our evaluation, the 3 health care systems participating in the study differed in many ways: Beth Israel Deaconess Medical Center (BIDMC) in Boston is a Harvard teaching hospital with approximately 113 hospital-based and community PCPs, serving approximately 90,000 patients, including over 50,000 patients registered to use *PatientSite*, a secure patient Internet portal based on a home-grown electronic medical record (EMR). Geisinger Health System (GHS) in rural northeastern Pennsylvania has approximately 175 PCPs serving about 300,000 primary care patients, with about 163,000 patients registered to use *MyGeisinger*, a patient portal based on the Epic EMR (Epic Systems Corp., Verona, WI). Harborview Medical Center (HMC) is a county-owned hospital in Seattle, WA managed by the University of Washington; HMC has a specific mission to care for the community's most vulnerable patients and is also the Disaster Control Hospital for Seattle and King County. HMC's two participating primary care clinics are staffed by approximately 65 faculty and fellows serving approximately 7,000 inner city and indigent patients, including about 2,000 patients with HIV. The internally-developed HMC patient portal, *HealthReach*, had been used only for research studies and not offered to the general HMC primary care patient population at the time we began this project. *HealthReach *was not integrated with HMC's EMR but could display information from ORCA, their clinical information system was based on the Cerner Millenium EMR platform (Cerner Corp., North Kansas City, MO) and other ancillary systems.

### Study design

**Figure 1:**
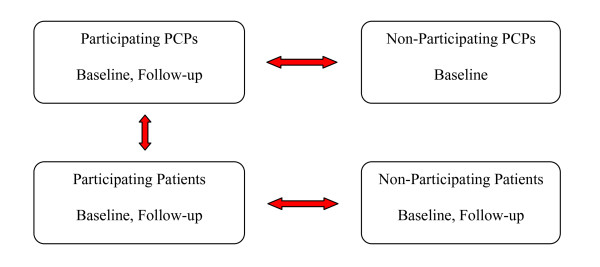
**Conceptual model showing primary planned comparisons among doctors and patients in their attitudes, experiences, and portal use before and after the implementation of OpenNotes**. Primary planned comparisons are indicated by red arrows.

In preparation for our OpenNotes demonstration and evaluation, we convened a team of investigators from across the U.S., including three (SR, JR, and TD) who had expertise in patient access to medical records and, specifically, doctors' visit notes. Informed by the literature and our own scientific experience related to patient and doctor attitudes toward open access to medical records [1-3], we developed a set of hypotheses about the potential impact of OpenNotes on primary care doctors and their patients, described in detail elsewhere [4]. In designing our test of the intervention, we specified 3 key areas where we hypothesized the greatest impacts would occur: beliefs and attitudes about patient accessible notes, use of the patient internet portals, and patient-doctor communication. We developed a quasi-experimental design that would allow us to perform a number of comparisons, portrayed in Figure 1, comparing doctors and patients, participants and non-participants, before, during, and after the one-year experience with OpenNotes.

We employed mixed methods, using qualitative data from semi-structured interviews and focus groups to inform survey instrument development and, at the end of the study, to provide a more in-depth assessment of patients' and doctors' experiences with OpenNotes. Using Creswell's typology, this approach is described as a sequential study involving qualitative data collection for instrument development, followed by quantitative data collection and subsequent qualitative data collection for triangulation purposes [5]. To structure our evaluation, we applied the RE-AIM framework [6] in order to capture as comprehensive a picture as possible of the impact of OpenNotes on patients, doctors, and health care systems. A scientific approach to assessing both the internal and external validity of real-world interventions, RE-AIM, is an acronym for: Reach, Effectiveness, Adoption, Implementation, and Maintenance. To address each of these elements of the intervention, we drew upon multiple data sources, including PCP and patient surveys, administrative and billing records, portal utilization data and electronic health records. The framework for our data collection and analysis is summarized in Table 1. Analyses directed to our primary and secondary study hypotheses will involve largely quantitative data from pre- and post-surveys of both doctors and patients, and also patient and doctor use of the Internet portals; subsequent qualitative analyses for secondary aims will utilize data obtained from patients' narrative survey information and patient and PCP focus groups.

**Table 1:** Summary of RE-AIM framework [6] for Evaluation of OpenNotes

Domain	Methods	Outcomes
Reach	Administrative data re: PCP characteristics and panel information; Portal use data; Surveys of patients/PCPs	PCP and patient demographics PCP workload, sessions, visits PCP portal statistics: number patients on portal, number of OpenNotes Patient portal statistics: registered users, logons

Effectiveness	PCP pre- and post- intervention survey, interviews Patient pre- and post- intervention survey PCP/patient portal data	PCP and patient preferences about continuation of OpenNotes PCP reported burden from OpenNotes Patient measures: Perceptions of Benefits/Risks of OpenNotes, ACES Quality of patient-doctor relationship subscale, Perceived Efficacy in Patient-Physician Interactions (PEPPI) short form questionnaire, PCP portal and telephone messages Patient portal: number of notes viewed

Adoption	System level data PCP practice data	Description of systems, PCP practices, proportions using OpenNotes PCP panel size, registered portal users

Implementation	Process data, PCP surveys and interviews, non-respondent surveys	PCP expectations, burden, barriers to participation; detailed descriptions of PCP experiences, comments, recommendations

Maintenance	PCP/system responses	Decision to continue or discontinue OpenNotes

We considered several study designs, but given the scope of the study and the involvement of 3 health systems, we identified a quasi-experimental, nonequivalent group, pretest-posttest approach as the one that would be most informative and efficient [7]. It was not feasible to use a traditional experimental approach because we knew that many providers would hesitate to be part of the first wave of participants for an intervention that would potentially impose further burdens on their already stretched schedules. Randomized allocation on the patient or provider level would have limited the study to only those PCPs who agreed to have their visit notes posted online and their patients and would have resulted in a much smaller number of patients who had access to their notes during the intervention period. Maximizing participation by allowing all willing PCPs to offer OpenNotes to their patient panels provided for the first large-scale effort to assess the impact of sharing visit notes electronically with patients.

### Survey instrument development

After developing our own list of expectations about the impact of OpenNotes [4] and before the one-year study began, we turned to patients and doctors in a series of focus groups to further explore attitudes and expectations about the intervention. The PCP focus group guide addressed communication between patients and PCPs, expected impact on PCP practice and workload, and impact on patients. Similarly, the patient focus group guide dealt with current communication and means of understanding regarding plan of care, and experiences and expectations about reading doctor's visit notes. Following 2 PCP focus groups (6 PCPs at GHS, and 8 at BIDMC) and 5 patient focus groups (11 patients at BIDMC, and 30 patients at HMC), three investigators (TD, EV, JW) reviewed transcripts of the recorded group sessions and identified common themes.

We developed survey questions based on the themes of PCP expectations about the impact on their practice and PCP and patient attitudes about benefits and risks of OpenNotes. Other than the set of questions specifically for PCPs about potential practice effects of OpenNotes, the PCP and patient surveys were designed in parallel to address similar questions about the anticipated benefits and risks of OpenNotes. In planning ahead for the one-year follow-up survey, we decided we would also include a set of questions about attitudes toward possible future modifications to OpenNotes that might further engage patients and their families in their care. We performed a series of tests of the pre-intervention surveys, first using paper questionnaires to ascertain the intent and clarity of the questions, then incorporating changes based on feedback from PCPs and patients, and we conducted additional testing in the online format to assess content and potential technical issues.

Concordant with the study's aims regarding the impact on attitudes, we developed 3 items to directly query patient respondents about whether or not they: a) thought OpenNotes was a good idea, b) would like to read their notes, and c) would be likely to share their notes with others. To explore these attitudes further, we developed three item sets, Perceptions of Benefits and Perceptions of Risks of OpenNotes on Patients, comprising 7 and 4 items, respectively, and a set of 4 items about Risks to PCP practices.

The survey questions regarding perceived patient benefits were as follows:

1. I would better understand my health and medical conditions.

2. I would better remember the plan for my care.

3. I would take better care of myself.

4. I would be more likely to take my medications as prescribed.

5. I would feel more in control of my health care.

6. I would be better prepared for visits.

7. Patients will trust me more as their doctor. (PCP survey only)

The questions addressing perceived patient risks were as follows:

1. I would worry more

2. I would be concerned about my privacy.

3. The notes would be more confusing than helpful.

4. It could make my doctor's job more difficult. (Patient survey only)

The PCP survey questions about the impact on their practice were as follows:

1. Patients will disagree with what I write in their visit notes

2. Patients will request changes to the content of visit notes

3. Patients will find significant errors in the notes

4. Patients will contact me or my practice with questions about their notes

The patient benefits and risk items were designed as parallel items on both patient and PCP surveys except where otherwise indicated. Response options were as follows: agree, somewhat agree, somewhat disagree, disagree; we added a "don't know" response option for patients. To compare PCP perceptions about anticipated and experienced impact of OpenNotes on their practices in the pre- and post-intervention surveys, respectively, we designed a set of questions addressing PCP documentation (content and process), communications with patients, potential impact on medical liability, and effects on patient care. In the baseline survey, we also included items about doctor and practice characteristics. For the post-intervention survey, to address our primary hypothesis, doctors and their patients in the intervention were specifically asked whether or not they would like OpenNotes to continue.

To measure patients' perceptions about patient-doctor communication, we used 2 standardized instruments. The Ambulatory Care Experiences Survey (ACES) was developed to survey patients about their experiences with health care providers, as a complement to existing health plan level strategies for assessing health care quality [8]. We used the 6-item ACES subscale assessing the quality of patient-provider interactions. Patients' self-confidence for communicating with doctors was measured using the validated Perceived Efficacy in Patient-Physician Interactions (PEPPI) scale [9]. This instrument was based on the previous work of Bandura regarding the assessment of self-efficacy, a motivator for health behavior [10,11]. The survey also included a set of questions regarding Internet use and sociodemographic and health characteristics (self-rated general health, education, race/ethnicity, and employment status). Lastly, in the follow-up survey, we included several open-ended questions, giving respondents opportunities to describe their experiences with OpenNotes and, if they did not read their notes, their reasons for not doing so.

For both patients and doctors, surveys were conducted online using SurveyGizmo (v2.0 & v3.0. Widgix, LLC dba SurveyGizmo, Boulder, Colorado). We required a response for all items except for demographic items, free text questions and questions within skip patterns. Based on our pilot tests, the PCP surveys were designed to be completed in less than 10 minutes, and the patient surveys in less than 20 minutes. The instruments are available from the authors upon request. Results regarding PCP and patients attitudes from the baseline survey were published elsewhere [12].

### Human subjects protections

Before the start of the study, all study protocols, data collection procedures and the intervention methods were approved by the institutional review boards of the 3 participating institutions, the BIDMC, the GHS and the University of Washington.

### Recruitment of primary care doctors

We began the study with the recruitment and enrollment of primary care doctors who would be willing to make their visit notes available to their patients through the Internet portal. OpenNotes doctors signed an informed consent prior to completing the baseline study surveys that preceded the OpenNotes intervention.

Invitations to join the intervention went to all primary care doctors (excluding trainees) practicing in BIDMC and affiliated primary care practices who were using both electronic health records (EHR) and the patient portal, *PatientSite*. PCPs were informed about the study through email announcements, presentations by study investigators, and informal discussions. We obtained the support of key clinical leaders within the institution who announced their endorsements on websites and through email communications with the PCPs. Similar recruitment strategies were employed at HMC and GHS, but because the GHS primary care practices are distributed geographically across a large area of rural central and northeastern Pennsylvania, the bulk of the recruitment contacts at GHS took place via email.

PCPs from the BIDMC and GHS primary care practices who chose not to offer any of their patients access to their visit notes online, referred to here as "non-intervention PCPs," were included in the study as the PCP comparison group. Their patients who were registered portal users comprised the comparison group referred to as "non-intervention patients." As no patient portal was available previously to patients at HMC, we did not have PCP or patient comparison groups at HMC.

### Recruitment of patients

After PCPs agreed to join the study, we sent them a list of their eligible patients and allowed them to exclude individual patients from the intervention with no requirement that they explain the exclusions. At BIDMC and GHS, patient eligibility was defined as having been registered on the patient Internet portal for at least 1 year before the start of the study. Following PCPs' exclusions, all patients registered on the patient Internet portals at BIDMC and GHS were included automatically as OpenNotes patients for the study, regardless of their portal use history or participation in study surveys. Prior to the start of the intervention, OpenNotes patients were sent a message through the portal informing them that their doctor was participating in OpenNotes, with an explanation of the study and a link to the OpenNotes website http://www.myopennotes.org that included detailed information about the study and the research team. The message included an invitation and a Web link to the pre-intervention survey.

We included two comparison groups of patients in our evaluation from BIDMC and GHS. The first group comprised patients of non-intervention PCPs who were registered portal users, referred to as "non-intervention patients." The second group included all patients of OpenNotes PCPs who were not registered portal users. This latter group was not approached to participate in surveys; their health care utilization and administrative data will be used in the evaluation. As noted above, there was no comparison group of HMC patients.

Prior to the OpenNotes Study, patients at HMC did not have access to the patient internet portal which was developed and implemented previously for research studies within the University of Washington system [2,13]. Therefore, the research team actively recruited HMC patients, many of whom were indigent or homeless but used the Internet through community venues. Eligibility required that a patient could communicate in English and had a current e-mail address. Some patients obtained new e-mail addresses in order to join the study. During the 4-month recruitment period, HMC staff used a multi-faceted approach that included letters of invitation mailed to all eligible patients with known addresses, active recruitment of a subset of eligible patients by telephone and in person recruitment of all eligible patients who came into the primary care clinics for a regularly scheduled clinic visit. Eligible patients who were approached about the study in the clinics were given an information flyer about the study and were invited to enroll after their clinic appointment or to join a bi-weekly group enrollment session. To enroll, HMC patients provided their email addresses, e-signed the study consent form, and then completed the online pre-intervention survey.

### OpenNotes: the intervention

Prior to OpenNotes, patients registered on the multifunctional GHS or BIDMC patient portals already had online access to their problem lists, medication records, and laboratory and radiology reports. In addition, the portals provided appointment scheduling and secure e-mail messaging among patients and their doctors and health care teams. As participants in the OpenNotes project, the PCP's at BIDMC or GHS could selectively exclude their patients, as described above, but they would be allowing all of their other eligible patients access to their visit notes if they were registered portal users at the time the study began in Spring, 2010.

At BIDMC and GHS, the OpenNotes intervention consisted of a simple addition to the existing menu of accessible records. Following a scheduled office visit, PCPs would record and sign their visit notes, at which point their patient would receive an automatically-generated e-mail invitation to read the visit note. Two weeks prior to a next scheduled visit to their PCP, patients also received an e-mail message suggesting that they review prior "OpenNotes" before coming to see their doctor. At HMC, patients did not have prior online access to their health records; therefore the intervention included new access not only to the visit notes, but also to other sections of their EHR including laboratory and radiology reports. Unlike the portals at BIDMC and GHS, the HMC portal was not interactive; patients could only view their records but could not schedule appointments or send messages to the healthcare team.

### Surveys of doctors and patients

We are using several data sources in the multi-faceted evaluation of OpenNotes, but the principal sources of information used to assess the study's primary outcomes are PCP and patient surveys conducted prior to, and then immediately following the one-year OpenNotes intervention. All surveys were conducted online using the web-based program, SurveyGizmo. Both intervention and non-intervention PCP's and their patients from the BIDMC and GHS were provided links to the surveys via survey invitation messages sent through the portals. Each respondent was given a unique study ID number assigned by the information technology (IT) staff of each health system. Survey data were downloaded from the password-protected SurveyGizmo databases into password protected files on the computer network at the study coordinating center at the BIDMC. At HMC, patients completed their surveys at the time of enrollment which took place in their HMC clinic.

### Electronic databases

In addition to the surveys, IT staff of each site assembled electronic data from multiple existing sources at the 3 sites, including the portal tracking systems, administrative databases and health care utilization databases. Using the unique study ID assigned to both intervention and non-intervention PCP's and patients at each site, we collected demographic and health characteristics, information about portal use, and health care utilization data for the year prior to the start of the intervention and for the one-year during which the visit notes were made available to intervention patients.

#### Patient characteristics

We used administrative data sources to obtain demographic information (age and sex) about PCPs and patients at each of the 3 sites.

#### Portal use data

We assessed portal use at each site through their electronic tracking systems. At BIDMC, HMC and GHS, the systems recorded the time and date of each patient portal login, each click into each section of the portal, including the OpenNotes section, and, with the exception of HMC, each message sent or received from within the portal. During the year of the intervention, we determined the total number of times patients entered the new OpenNotes sections of their portal records.

#### Health care utilization

Patient utilization measures, determined from billing records, included counts of visits to PCP's and their practices, hospitalizations, and emergency room visits.

## Results

**Figure 2:**
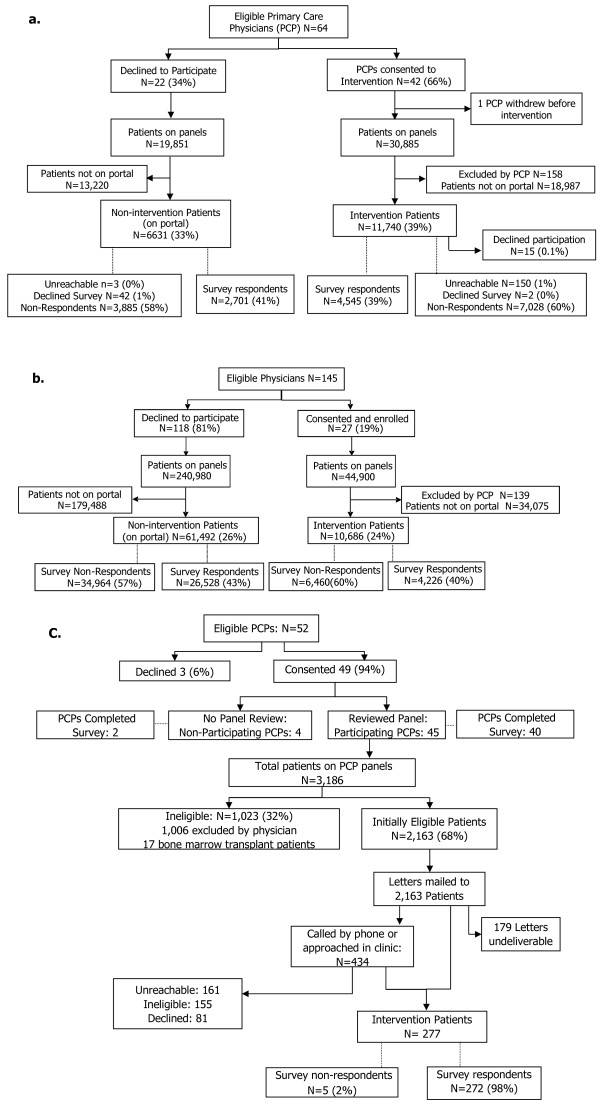
**(A) BIDMC PCP and Patient Recruitment and Enrollment, (B) GHS PCP and Patient Recruitment and Enrollment, (C) HMC PCP and Patient Recruitment and Enrollment**.

Rates of PCP participation in the OpenNotes intervention varied widely among the 3 study sites. At GHS, 27 (19%) out of 145 invited PCPs enrolled; at BIDMC, 42 (66%) out of 64 invited PCPs enrolled; at HMC, 45 (87%) out of 52 invited PCPs enrolled in the intervention. Invited PCPs who declined participation at BIDMC and GHS were included in the control group of non-participating PCPs (GHS, n = 118; BIDMC, n = 22). The recruitment and enrollment of PCPs and their patients at the 3 participating sites are shown in Figure 2A-C.

From among their patient panels, intervention PCPs at BIDMC and GHS excluded 158 and 139 patients from participating, respectively, or 1.3% and 1.4% of eligible patients. At HMC, the entire panel of adult patients managed by each intervention PCP was initially eligible for the study, and the PCPs excluded 1,006, or 32% of their eligible patients. Though we did not collect reasons for exclusion, the HMC patient population includes many individuals with psychiatric diagnoses, current substance abuse, or other severe medical conditions (eg. bone marrow transplant).

At the BIDMC, 11,740 patients (39%) from the panels of participating PCPs were registered portal users and eligible to participate in the intervention. Only 15 patients (0.1%) requested to not be included in the intervention after receiving their notification letter about OpenNotes and their invitation to complete the survey. 4,545 intervention patients (39% of those invited) submitted the optional online survey prior to the start of the intervention. On the panels of the non-participating PCPs, 6,631 patients were registered on the portal and invited to complete the online survey; 2,701 (41%) submitted the survey.

Within GHS, of the nearly 45,000 patients on the panels of participating doctors, 10,686 patients (24%) were registered on the *MyGeisinger *portal. Of these, 4,226 (40%) responded to the online survey. Among the PCPs who did not participate in the intervention, 61,492 patients were registered on the portal and invited to participate in the survey. Of these, 26,528 patients (43%) submitted the online survey.

At HMC, patient recruitment was individualized because there was no previous web portal available to patients. For the initial recruitment, invitation letters were mailed to 2,163 eligible patients; however, 179 patients (8%) did not have a viable mailing address or their letters were returned undeliverable. The second step in the recruitment process involved telephoning patients from the PCPs' panel lists, or directly approaching patients in the clinic before their clinical appointment. Of 434 patients contacted by phone or approached in the clinics, 155 were ineligible primarily due to language barriers (n = 34) or lack of an email address (n = 113). Eligible and interested patients were asked to complete the survey online as part of the study enrollment. Five patients (2% of enrollees) experienced technical problems submitting their surveys which, as a result, were not recorded. There were 272 HMC patients who completed the online survey.

### Characteristics of PCPs

Doctors who participated were somewhat younger than non-participating PCPs at BIDMC and HMC (Table 2); there were higher proportions of men among the participating PCPs; and participating PCPs generally had smaller panels, compared to non-participating PCPs. However, none of the observed differences between groups were statistically significant.

**Table 2:** Characteristics of participating and non-participating primary care physicians (PCPs) according to study site*

	BIDMC PCPs		GHS PCPs		HMC PCPs	
	
Characteristics	Participating (n = 42)	Non-participating (n = 22)	Participating (n = 27)	Non-participating (n = 118)	Participating (N = 45)	Non-participating (N = 4)
Age (mean ± S.D.)	47.3 ± 9.6	50.8 ± 6.7	49.7 ± 8.2	49.9 ± 9.4	42.5 ± 6.9	49.8 ± 5.4

Sex (female)	20 (47.6%)	11 (50.0%)	6 (22.2%)	38 (32.2%)	26 (57.8%)	3 (75.0%)

Panel size:						
< 500	18 (42.9%)	7 (31.8%)	4 (14.8%)	9 (7.6%)	45 (100%)	4 (100%)
500-999	14 (33.3%)	7 (31.8%)	5 (18.5%)	12 (10.2%)	0	0
1000-1999	8 (19.0%)	6 (27.3%)	7 (25.9%)	42 (35.6%)	0	0
≥ 2000	2 (4.8%)	2 (9.1%)	11 (40.7%)	55 (46.6%)	0	0

* Differences between participating and non-participating PCPs tested using t-tests for continuous measures and Chi-square tests for categorical measures. No significant differences were observed between participating and non-participating PCPs at any of the 3 sites

### Characteristics of patients

On average, both participating and non-participating patients were approximately aged 50 years at each of the 3 sites (Table 3). Approximately 60% were female in the Boston and Pennsylvania sites, with a slightly higher percentage of women in the non-participating patient groups. In Seattle, 24% of participating patients were women.

**Table 3:** Characteristics* of participating and non-participating patients according to study site

Characteristics	BIDMC Patients		GHS Patients		HMC Patients
	
	Participating	Non-participating	Participating	Non-participating	Participating
Age (mean ± S.D.)	49.4 ± 13.7	51.3 ± 14.3	49.9 ± 15.6	49.4 ± 15.3	48.9 ± 11.0

Sex (% female)	57.4%	59.6%	58.0%	61.8%	23.7%

*Information derived from administrative records of each site

## Discussion

To our knowledge, this is the first extensive evaluation of a large-scale implementation of online patient access to doctors' visit notes through patient Internet portals. Our recruitment process demonstrates that substantial numbers of primary care providers are willing to engage in a year-long trial of this potentially disruptive intervention. Large numbers of primary care patients have had access to sections of their health records online for several years at the BIDMC and GHS. Although the addition of the office visit notes to these readily accessible online records seemed like a relatively minor change, many doctors voiced concerns in advance of the trial and even opposition to this next step in the evolution of transparency in healthcare.

Moving beyond small scale and qualitative studies of the impact of patient access to medical records, we designed the study to involve 3 large institutions with distinctly different patient populations. We intended to enroll as many PCPs and patients as possible and to capture as much quantitative and qualitative information as possible in our evaluation. Thus, in planning the evaluation and in giving careful consideration to our choice of research design and methods, we sought to collect new information and to maximize use of existing data sources. We used an online survey to address our key hypothesis about whether or not PCPs and patients would want to continue OpenNotes at the conclusion of the trial. To explore this further, the survey included questions assessing attitudes and perceptions about the impact of OpenNotes. We made use of both administrative data and data from portal tracking systems to address our secondary hypotheses, to cross-validate survey responses from PCPs and patients with actual portal use before and during the intervention period.

Our selection of study sites was intended to create a diverse pool of PCP practices and their patients. Although the majority of PCPs from the Boston (66%) and Seattle (87%) sites agreed to join the intervention, in Pennsylvania, a much smaller proportion of providers (19%) from these rural practices enrolled. On the other hand, panel sizes in Pennsylvania were generally much larger than the other 2 academic-affiliated institutions, and large numbers of patient from GHS (more than 10,000) were thereby enrolled in the intervention. Unlike the other sites, recruitment of the geographically dispersed GHS providers relied more on written communication than in-person discussions and presentations about the study, a factor that may have contributed to lower PCP participation.

In conclusion, many primary care physicians were willing to participate in this new intervention despite concerns of a potential burden to their practices related to patient inquiries about their notes. This positive response attests to the feasibility of conducting a large scale, multisite study of the impact of an intervention using electronic health records and Internet patient portals. Extensive electronic data resources will make it possible to conduct a thorough multi-faceted evaluation of PCP and patient level factors to determine the impact of giving patients online access to their physician's office visit notes. In addition, the availability of online survey technology enabled us to collect extensive information about attitudes and expectations before notes were made readily available, and about actual experiences after using such notes for at least one year. Results from this evaluation will inform providers, policy makers, and patients who contemplate such changes at a time of exploding interest in transparency, patient safety, and improving the quality of care.

## Abbreviations

ACES: Ambulatory Care Experiences Survey; BIDMC: Beth Israel Deaconess Medical Center, Boston, MA; GHS: Geisinger Health System, Danville, Pennsylvania; HMC: Harborview Medical Center, Seattle, WA; PCPs: Primary care physicians; PEPPI: Perceived Efficacy in Patient-Physician Interactions scale; RE-AIM: Reach, Effectiveness, Adoption, Implementation, and Maintenance.

## Competing interests

The authors declare that they have no competing interests.

## Authors' contributions

All authors contributed to the conception, design, data acquisition and analysis, interpretation of data and each has been involved in drafting or revising the manuscript. All authors have provided final approval of the published version of the paper.

## Pre-publication history

The pre-publication history for this paper can be accessed here:

http://www.biomedcentral.com/1472-6947/12/32/prepub
